# Sex‐Dependent Carry‐Over Effects Between Physiological State and Reproduction in a Passerine Species

**DOI:** 10.1002/ece3.71816

**Published:** 2025-07-20

**Authors:** G. Szabó, N. Boross, G. Hegyi, M. Herényi, M. Jablonszky, D. Kötél, K. Krenhardt, G. Markó, G. Nagy, B. Rosivall, E. Szász, E. Szöllősi, S. Zsebők, J. Török, M. Laczi

**Affiliations:** ^1^ Behavioural Ecology Group, Department of Systematic Zoology and Ecology ELTE Eötvös Loránd University Budapest Hungary; ^2^ Doctoral School of Biology, Institute of Biology ELTE Eötvös Loránd University Budapest Hungary; ^3^ Lendület Ecosystem Services Research Group Institute of Ecology and Botany, HUN‐REN Centre for Ecological Research Vácrátót Hungary; ^4^ HUN‐REN Balaton Limnological Research Institute Tihany Hungary; ^5^ HUN‐REN‐ELTE‐MTM Integrative Ecology Research Group ELTE Eötvös Loránd University Budapest Hungary; ^6^ Department of Zoology and Ecology Hungarian University of Agriculture and Life Sciences Gödöllő Hungary; ^7^ Evolutionary Ecology Research Group Institute of Ecology and Botany, HUN‐REN Centre for Ecological Research Vácrátót Hungary; ^8^ Department of Plant Pathology Institute of Plant Protection, Hungarian University of Agriculture and Life Sciences Budapest Hungary; ^9^ Department of Reference Sample Analysis Hungarian Institute for Forensic Sciences (HIFS), Institute of Forensic Genetics Budapest Hungary; ^10^ The Barn Owl Foundation Orosztony Hungary

**Keywords:** breeding success, collared flycatcher, H/L ratio, haematocrit, reproductive role, stress

## Abstract

Carry‐over effects (COEs) occur when an event in a life stage of an individual is affected by the experience, physiological state or reproduction of a previous life stage. COEs are insufficiently explored with regard to the connections between physiological state and reproduction in animals with seasonal iteroparity and short lifespans. We investigated within‐individual temporal changes in haematocrit (an indicator of oxygen‐carrying capacity and energetic demand) and heterophil granulocyte‐to‐lymphocyte (H/L) ratio (indicator of stress levels and health state) within and between breeding seasons, and COEs between haematocrit, H/L ratio, and reproduction (breeding onset, clutch size, brood size and the number of prefledglings) in a short‐lived passerine, the collared flycatcher ( *Ficedula albicollis* ). Haematocrit varied across years and showed moderate repeatability overall, with higher repeatability observed in females. In males, haematocrit declined between the courtship and the nestling‐rearing period. Lower haematocrit during nestling rearing was associated with higher brood size in males, suggesting a trade‐off between self‐maintenance and parental effort. While H/L ratio did not fluctuate across years, in females, increased reproductive effort in the previous year correlated with higher H/L ratios in the subsequent year, indicating physiological costs of reproduction. Additionally, females with higher H/L ratio during nestling‐rearing had lower fledging success in the following year, suggesting long‐term fitness consequences of stress. Our findings highlight that COEs between physiology and reproduction in the short‐lived collared flycatcher manifest differently in the two sexes, likely due to distinct reproductive roles and energetic constraints.

## Graphical abstract

We investigated sex‐specific carry‐over effects (COEs) between physiological state and reproduction in the short‐lived, seasonally breeding collared flycatcher ( *Ficedula albicollis* ). Our results reveal that COEs manifest differently between sexes: in females, past reproductive effort and stress levels predicted future physiological condition and reproductive outcomes, while no such patterns were observed in males. These findings provide novel insights into the physiological costs of reproduction and highlight the importance of sex‐specific approaches in ecophysiological studies.

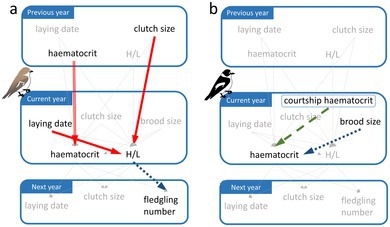

## 1 Introduction

In seasonal environments, the annual life cycle of vertebrates can have distinct, sequential stages, such as reproduction, moulting and dispersal. These stages are physiologically interconnected, so events in one life stage can have an effect on subsequent stages (Harrison et al. 2011). When an individual's previous experience and physiological state affect its performance in a following stage, carry‐over effects (COEs) occur (O'Connor et al. 2014). Although physiological state is well established as an important determinant of reproductive success, relatively little is known about the specific COEs linking physiology and reproductive success (Harrison et al. 2011). Furthermore, COEs can be sex‐dependent (Saino et al. 2017), even between physiological condition and reproductive success (Saino et al. 2012).

Reproduction (e.g., egg‐laying, incubation, provisioning) imposes high physiological demands on birds (e.g., Vleck et al. 2000; Moe et al. 2002; Williams et al. 2004; Bókony et al. 2009), so reproductive success could be linked with multiple physiological traits, including haematocrit (Crossin et al. 2017; Fronstin et al. 2016), corticosterone (CORT; for review, see Bonier et al. 2009) and leukocyte profiles, particularly heterophil granulocyte‐to‐lymphocyte (hereafter H/L) ratio (Ochs and Dawson 2008). These physiological traits reflect different aspects of the individual's physiological state; hence, they can be connected with reproduction in different ways.

Haematocrit is the relative volume of red blood cells in whole blood, which reflects the oxygen‐carrying capacity and energetic demand of an individual. Low haematocrit can indicate an energy deficit limiting erythropoiesis due to prolonged physical exertion or malnutrition (Owen and Moore 2006; Campbell 1994), while high haematocrit can reflect elevated oxygen‐carrying capacity (Saino et al. 1997) or dehydration (Vleck et al. 2000). Both excessively high and low haematocrit can impair oxygen‐carrying capacity, as low haematocrit indicates fewer red blood cells carrying oxygen, and high haematocrit increases blood viscosity, reducing oxygen‐carrying efficiency (Birchard 1997). Multiple factors can mediate the relationship between reproduction and haematocrit, for example increased parental load and need for elevated oxygen‐carrying capacity (Hõrak, Ots, and Murumägi 1998; Fowler and Williams 2017). In addition, reproductive effort and haematocrit have a bidirectional relationship, as haematocrit can affect breeding effort (Fronstin et al. 2016) and vice versa (Fowler and Williams 2017; Hõrak, Ots, and Murumägi 1998).

Leucocyte profiles are widely used and easily measured descriptors of animal immune function (Norris 2000). Lymphocytes (i.e., T and B cells) are part of the adaptive immune system, producing immunoglobulins to recognise pathogens, while heterophils use phagocytosis to fight pathogens as the first line of defence. While the H/L ratio is not a measure of immune response (Davis et al. 2008), it can reflect the relative readiness to fight infection via heterophils (through injury) versus lymphocytes (viral and bacterial infection; Johnstone et al. 2012). Similarly to the case of haematocrit, the energy allocation into innate and adaptive immune response can create a trade‐off between reproduction and the H/L ratio (Martin et al. 2006). The H/L ratio is linked not only to the immune system but also to stress as lymphocytes leave (i.e., lymphopenia), and heterophils enter circulation in response to elevated CORT levels (i.e., heterophilia) (Davis et al. 2008; Dhabhar et al. 1996). Both CORT and the H/L ratio are indices of physiological stress. However, CORT levels change rapidly (within 10–30 min) under acute stressors, so the H/L ratio may be better suited for assessing chronic stress (Davis and Maney 2018). Experimental studies in different species have revealed a positive relationship between parental effort and the H/L ratio in great tits ( *Parus major* ), blue‐footed boobies ( *Sula nebouxii* ) and pied flycatchers ( *Ficedula hypoleuca* ) (Hõrak, Ots, and Murumägi 1998; González‐Medina et al. 2015; Ilmonen et al. 2003), suggesting that this pattern can be consistent across long‐ and short‐lived birds.

Even though the connections between reproduction and physiological state are well‐explored, only a few studies have examined whether haematocrit and physiological stress predict future reproductive performance or whether past reproduction predicts future physiological state. A study on house wrens ( *Troglodytes aedon* ) suggests that nestling haematocrit predicts long‐term survival, thus lifetime reproductive success, as individuals with intermediate haematocrit values exhibit greater longevity (Bowers et al. 2014), while in two long‐living species (black‐browed albatrosses [ *Thalassarche melanophris* ] and grey‐headed albatrosses [ *T. chrysostoma* ]) haematocrit levels during breeding did not affect future breeding decisions (Crossin et al. 2017). In terms of physiological stress, H/L ratio during incubation did not affect whether common eiders ( *Somateria mollissima* ) return to breed the next year (Hanssen et al. 2003). Postbreeding CORT levels of common eiders (Steenweg et al. 2022; Harms et al. 2015) and grey‐headed albatrosses (Crossin et al. 2017), and prebreeding CORT levels of black‐legged kittiwakes ( *Rissa tridactyla* ) had no direct impact on future reproductive output (Léandri‐Breton et al. 2024). However, black‐browed albatrosses and giant petrels (*Macronectes* spp.) with higher postbreeding CORT levels were more likely to defer breeding in the subsequent breeding season (Crossin et al. 2013, 2017). Postbreeding CORT levels affect the timing of breeding‐site arrival of eiders (Harms et al. 2015), but not in kittiwakes (Schultner et al. 2014). These studies show that CORT can affect future reproduction. In Cory's shearwaters (*Calonectris borealis*) (Ramos et al. 2018) experimentally reduced reproductive success resulted in lower postbreeding CORT levels.

At this point, it is necessary to highlight that studies on haematocrit, stress physiology in relation to reproductive COEs in birds that we know of focused on relatively long‐lived species, except for one study on house wrens (Bowers et al. 2014). Therefore, research on short‐lived species is warranted to clarify the overall picture, especially if we consider that some of the parameters, for example H/L ratio, can be linked to longevity (Minias 2019), and different indices of physiological stress can be linked to certain fitness components (e.g., survival) at different time scales, even within a species (Maness et al. 2023). Since fitness is linked to haematocrit and stress physiology, it would have crucial importance to reveal the within‐individual temporal change and also the repeatability of the aforementioned physiological measures at different time scales. However, only a handful of studies addressed these questions, focusing on the temporal patterns within the reproductive season (haematocrit: Boross et al. 2012; Wagner et al. 2008; Williams et al. 2004; H/L ratio: Kulaszewicz et al. 2017; CORT: Fletcher et al. 2018) and between years (haematocrit: Potti 2007; Boross et al. 2012; H/L ratio: Ochs and Dawson 2008; CORT: Angelier et al. 2009, 2010; Cockrem et al. 2009; Ouyang et al. 2011). In general, there is a need to systematically extend research on physiology and reproduction beyond a single breeding period in birds (Marra et al. 2015).

The collared flycatcher ( *Ficedula albicollis* ) is a trans‐equatorial migrant passerine. In this species, the reproductive investment could have an effect on the postbreeding autumn migration and the following spring migration schedule (Briedis et al. 2018). This species‐specific feature suggests a significant role of COEs as migration patterns can influence future physiology (Marra et al. 1998) and reproductive success, and these patterns can be sex‐dependent (Saino et al. 2017). However, little is known about the underlying physiological mechanism. Here, we aimed to examine the COEs between haematocrit, H/L ratio and reproduction of the collared flycatcher, which is, in contrast to most previous study subjects, a short‐lived bird (Mourocq et al. 2016). In our study population, we previously found that haematocrit declined from the courtship to the nestling‐rearing period in males, and it was repeatable across breeding seasons (Boross et al. 2012). In a closely related species, the pied flycatcher, brood size reduction resulted in reduced H/L ratio in males (Ilmonen et al. 2003), and prefledging H/L ratio was lower in individuals that returned to the breeding population in later years (Lobato et al. 2005). Based on these findings, our aim was to reveal the patterns of COEs with regard to H/L ratio, haematocrit and reproduction. In detail, we predicted that haematocrit decreases between breeding phases and that the current physiological state is negatively associated with the magnitude of breeding effort. We further predict that higher reproductive effort in the previous year leads to worse physiological state in the current breeding season, and individuals in better current physiological state will perform better with respect to reproduction in the subsequent breeding season. Also, we predicted that haematocrit during nestling rearing is highly repeatable between years.

## 2 Materials and Methods

### 2.1 Study Area and Species

Between 2013 and 2019, we collected our data from a collared flycatcher population breeding in a nest box plot located in an oak‐dominated ( *Quercus cerris* , *Q. petraea* ) woodland in the Pilis–Visegrádi Mountains, Duna‐Ipoly National Park, Hungary (47°43′ N, 19°01′ E; see details in Török and Tóth 1988). After migration, collared flycatchers return to the breeding grounds in mid‐April. Females usually lay 5–7 eggs and raise one brood per year (e.g., re‐nesting may occur after a previous failed attempt). Females incubate for 10–11 days, and both parents partake in nestling rearing. In the study population, 6% of males are polygynous, who put significantly lower effort into chick rearing in their secondary nests (Garamszegi et al. 2004). The average lifespan of breeding females in our population (calculated from 1987 to 2023) is 2.5 years, and 2.4 years for breeding males (upper quartiles are 3–9 years for both sexes).

### 2.2 Field Methods

We checked nest boxes every 4–5 days to determine the laying date of the first egg. We recorded the clutch size (i.e., number of eggs), the brood size (i.e., nestling number at the time of capture of their parents, see later) and the fledgling number (number of prefledglings, i.e., nestlings reaching 13 days of age; hereafter, fledgling number). To account for interannual shifts of the breeding season, we calculated the laying date relative to the median value of laying dates across all clutches each year (hereafter, laying date). We chose the median over the mean due to the positively skewed distribution of laying dates. Clutch size, brood size and fledgling number were strongly and negatively correlated with laying date. To adjust for these correlations, we performed ordinary least squares linear regressions, using laying date as the predictor variable, and calculating residuals from the fitted regression line for each reproductive variable in each year (Table A1: Appendix S3). Therefore, in the subsequent analyses, the reproductive variables were represented by their residuals. Some individuals were breeding in multiple years (the number of individuals caught in pairs of years, female/male: 2013 and 2014: 36/26; 2014 and 2015: 47/48; 2015 and 2016: 76/80; 2016 and 2017: 87/86; 2017 and 2018: 104/88; 2014 and 2016: 27/28; 2016 and 2018: 50/52).

### 2.3 Blood Samples

We collected blood samples in 2014–2016 and 2018 (we did not take blood samples in 2017 due to technical reasons) in the nestling‐rearing phase, when their nestlings were 8–10 days old and marked them using numbered rings. We took blood samples (~40 μL) from the brachial vein into heparinised capillaries in 2014–2016 and 2018 (sample sizes females/males: 2014: 60/39; 2015: 95/55; 2016: 159/132; 2018: 86/79). Additionally, we captured 14 males (2015:1; 2016:2; 2018: 11) during the courtship period, 36.6 ± 3.8 days (mean ± SD) before their recapture during nestling rearing. We centrifuged the blood collected in capillaries within 2 h of sampling (10 min, 10,000 *g*). We measured plasma and total blood lengths in the capillary to the nearest millimetre and calculated haematocrit (%) using the formula: haematocrit = (total blood length—plasma length)/total blood length × 100, as the length of a liquid in a capillary is directly proportional to its volume.

We prepared blood smears to assess H/L ratio in 2015 (females/males: 35/27) and 2016 (females/males: 112/94). We air‐dried and fixed blood smears in absolute methanol for 2 min, and stained them with a 10% Giemsa solution for 50 min. All blood smears were examined by GS, using a microscope (Zeiss Axioskop) at 1000× magnification. We recorded the field of view with a camera connected to AnalySIS Software 5.0 (Olympus Soft Imaging Solutions). We counted lymphocytes and heterophil granulocytes across 200 fields of view per smear (Owen and Moore 2006). We calculated the H/L ratio by dividing the number of heterophils by the number of lymphocytes.

### 2.4 Statistical Analyses

We used generalised linear models (GLMs) and generalised linear mixed‐effects models (GLMMs) for our analyses. We were expecting sex‐dependent patterns, but could not include the pairwise interaction between sex and other predictors in most models, as most birds (70+%) were breeding pairs, and we would have to add nest ID as a random effect, which would result in overfitted models with convergence problems. Therefore, we analysed males and females in separate models. We excluded (i) data of socially polygynous males (2018: *n* = 3), (ii) data from birds participating in experiments that may have affected their physiology or breeding variables (females/males: 2015: 12/7; 2016: 27/26) and (iii) reproductive data after a nest was partially or fully predated (females/males: 2013: 1/1; 2014: 5/6; 2015: 2/4; 2016: 9/10; 2018: 2/1; 2019: 4/4).

For all models, we applied a BIC‐based selection approach. We chose the candidate model with the lowest BIC score after removing explanatory variables one by one. If the BIC value of multiple models was within 2, we chose the simplest model. We did not remove main effects before removing their interactions. We repeated this until we reached an optimal model fit. We used Wald *χ* ^2^ statistics to test the significance of the fixed effects. The highest variance inflation factor was 3.0 in all analyses, suggesting that multicollinearity had a low impact on the results (Zuur et al. 2010). Correlation matrices between predictors are in Tables C1 and C2: Appendix S3. We prepared all analyses and data visualisation using R 4.3.3 (R Core Team 2024). We constructed GLMMs using glmmTMB 1.1.9 (Brooks et al. 2017) package. We used car 3.1‐2 (Fox and Weisberg 2019), DHARMa 0.4.6 (Hartig 2022) and emmeans 1.10.3 (Lenth 2024) packages for model diagnostics and evaluation. We used rptR 0.9.22 (Stoffel et al. 2017) package with 1000 bootstraps and 1000 permutations to estimate repeatability and confidence intervals. We used tidyverse 2.0.0 (Wickham et al. 2019) package for data preparation.

### 2.5 Among‐ and Within‐Year Patterns of Haematocrit and H/L Ratio

To assess within‐individual variation in haematocrit across consecutive years (2014–2015 and 2015–2016), we calculated repeatability for the entire data set and separately for males and females, too. Additionally, since population‐level haematocrit varied across years, we recalculated repeatability using haematocrit values standardised for year (mean = 0, SD = 1), separately for males and females. The same procedure was applied to individuals recaptured with a 1‐year gap (2014–2016 and 2016–2018), in order to assess repeatability in a longer time period. We compared the haematocrit of males between the courtship and nestling phases with Student's *t*‐test for paired samples. We did not calculate within‐individual repeatability for H/L, due to the limited sample size (only eight individuals with consecutive‐year data). However, in 16 blood smears, we counted leukocytes twice on nonoverlapping areas to assess repeatability of H/L ratio, and it was highly repeatable (*R* = 0.70, LRT *p* < 0.001). In these cases, we used the mean H/L value for further analyses.

We used GLMMs to explore between‐year patterns of haematocrit (Table B1 in Appendix S3) and H/L ratio (Table B2 in Appendix S3) of all sampled birds during the nestling phase in separate models. Fixed factors were year, sex and their interaction, and bird identity was included as a random effect. We initially evaluated models with Gaussian distribution with identity function for haematocrit and H/L ratio and chose this for haematocrit, while we could not reach an optimal model fit for H/L ratio based on the distribution of the residuals. We found a better goodness of fit for the model with H/L ratio as the response variable when using gamma distribution and log‐link function, indicated by the right‐skewed distribution of the residuals and a lower Bayesian Information Criterion (BIC) value. Furthermore, we performed post hoc pairwise comparisons between years and between sexes using Tukey's method for multiple testing.

### 2.6 Relationship Between Current Reproduction and Current Physiology

We used GLMMs with Gaussian distribution and identity function to analyse how reproductive effort in the current year was associated with current physiology. We included year, current laying date, clutch size, brood size and the pairwise interactions with year of the reproductive variables in the model as fixed effects. We would have liked to include exact age as a covariate; however, we only know the ages of 20% of females and 50% of males. Bird identity was included as a random factor since many individuals were captured in multiple years (sample sizes: female/male: 223/214; Tables B3.1 and B3.2 in Appendix S3). In the case of H/L ratio and current reproduction, we had much less data from 2015 than from 2016 (2015: female/male: 14/21, 2016: 64/68), so we used data from only 2016. Therefore, we neither included bird identity nor year or the pairwise interactions with year in the models (Tables B4.1 and B4.2 in Appendix S3).

### 2.7 Relationship Between Previous Reproduction and Current Physiological State

We used generalised linear models (GLMs) with Gaussian distribution and identity function to analyse how reproductive effort in the previous year was associated with current physiology. We included previous laying date and previous clutch size, and current laying date and brood size (to control for the effects of current reproduction on current physiology) as predictors in the models (H/L ratio sample sizes, female/male: 2016: 12/14, 2015: 10 altogether; Tables B5.1 and B5.2 in Appendix S3). We would have preferred using fledgling number from the previous year, as it reflects the amount of nestlings the parents reared (similarly to current brood size), but that would have drastically decreased the sample sizes; hence, we chose clutch size. The models with haematocrit included the interactions of previous laying date, clutch size with year (factor), and current laying date and brood size (sample size: female/male: 38/31, Tables B6.1 and B6.2 in Appendix S3). We also ran a model including previous haematocrit, which gave the same results shown in Section B7 (Tables B7.1 and B7.2 in Appendix S3): Appendix S3.

### 2.8 Relationship Between Current Physiology and Future Reproduction

We used GLMs with Gaussian distribution and identity function to analyse how current physiology is linked to future reproduction. We ran separate models for laying date, clutch size and fledgling number of next year as response variables, and included haematocrit and H/L ratio as fixed effects. Only six females and seven males had both haematocrit and H/L from 2015, so we used physiological data only from 2016 in the models (sample sizes female/male, laying date: 18/18; Tables B8.1 and B8.2 in Appendix S3; clutch size: 17/18; Tables B8.3 and B8.4 in Appendix S3; fledgling number: 15/16; Tables B8.5 and B8.6 in Appendix S3). We ran the above models without the H/L ratio, thus increasing the sample size (Tables B9.1–B9.6 in Appendix S3). This model showed the same results; see details in Section B9: Appendix S3. For details about the models, see Appendix S3.

## 3 Results

### 3.1 Among‐ and Within‐Year Patterns of H/L and Haematocrit

**FIGURE 1:**
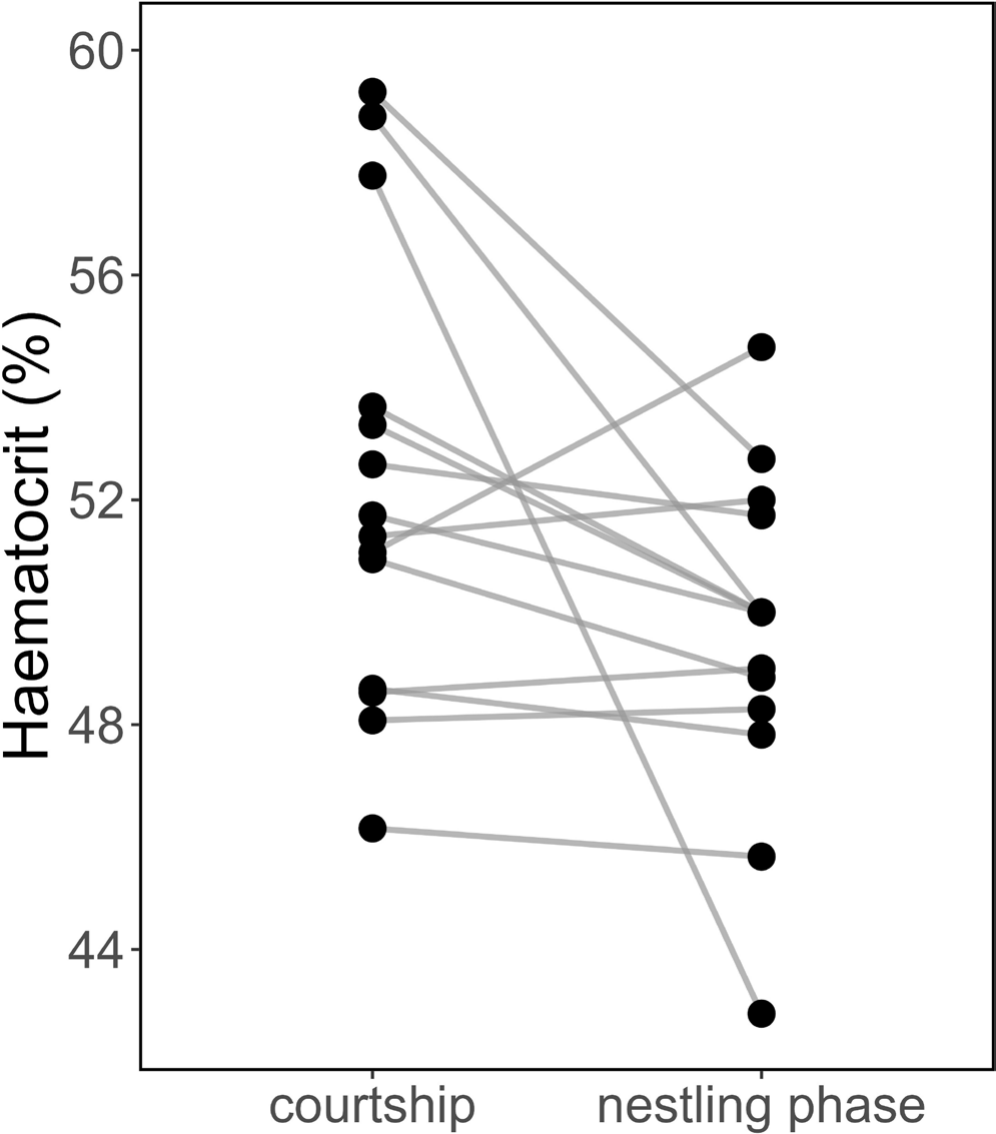
Decrease of haematocrit in male collared flycatchers between courtship and nestling‐rearing period. The dots show the haematocrit values, and the lines connect the values of the same individuals.

Year‐standardised haematocrit was moderately repeatable across consecutive years (*n* = 36, *R* = 0.42, likelihood ratio test (LRT) *p* = 0.018), particularly in females (*n* = 17, *R* = 0.51, LRT *p* = 0.026); however, it was not repeatable when calculated with raw values (all *R* < 0.26, all LRT *p* > 0.18), or with a 1‐year gap (all *R* < 0.30, all LRT *p* > 0.13, Table 1). In males, haematocrit decreased between the courtship and nestling‐rearing period (*n* = 14, *t* = 2.19, *p* = 0.047, Figure 1). We found that the H/L ratio did not differ between years or sexes (the optimal model was the null model), whereas the haematocrit differed between both years and sexes (year: *n* = 485, *χ* ^2^ = 38.0, *p* < 0.001; sex: *n* = 485, *χ* ^2^ = 5.5, *p* = 0.019), with females generally having higher haematocrit than males (Table 2). Post hoc pairwise analyses revealed that haematocrit was higher in 2014 and 2018 compared to 2015 and 2016 (2014–2015: *p* < 0.001, 2014–2016: *p* = 0.004, 2014–2018: *p* = 0.94, 2015–2016: *p* = 0.055, 2015–2018: *p* < 0.001, 2016–2018: *p* = 0.002).

**TABLE 1:** Repeatabilities (*R*) and 95% confidence intervals (CI) of haematocrit within individuals (*N*), between consecutive years and with a 1‐year gap.

	Female			Male			Together		
	*N*	*R*	95% CI	*N*	*R*	95% CI	*N*	*R*	95% CI
Consecutive years									
Year‐standardised haematocrit	**17**	**0.51**	**0.02–0.80**	19	0.28	0–0.70	**36**	**0.42**	**0.08–0.67**
Haematocrit	17	0.13	0–0.68	19	0.14	0–0.61	36	0.17	0–0.51
1‐year gap									
Year‐standardised haematocrit	10	0.06	0–0.60	11	0.26	0–0.71	21	0.15	0–0.55
Haematocrit	10	0.25	0–0.68	11	0.30	0–0.73	21	0.26	0–0.6

*Note:* The standardisation of haematocrit is done by year and separately for the sexes (mean = 0, SD = 1). The significant repeatabilities are in bold.

**TABLE 2:** Descriptive statistics of physiological and reproductive variables.

Year	Sex	Haematocrit		H/L		Number of eggs per nest		Number of nestlings at the time of capture		Number of fledglings	
		*n*	Mean ± SD	*n*	Mean ± SD	*n*	Mean ± SD	*n*	Mean ± SD	*n*	Mean ± SD
2014	Male	31	49.3 ± 2.2	0		41	6.5 ± 0.7	41	5.9 ± 0.9	27	5.7 ± 1.1
	Female	34	49.7 ± 2.8	0		45	6.5 ± 0.7	45	5.9 ± 0.9	29	5.9 ± 1.0
2015	Male	40	47.0 ± 2.6	14	0.21 ± 0.12	45	5.9 ± 1.1	40	4.9 ± 1.6	28	4.7 ± 1.6
	Female	48	47.9 ± 2.8	23	0.27 ± 0.19	55	6.1 ± 0.8	48	4.9 ± 1.6	36	4.6 ± 1.5
2016	Male	95	47.8 ± 2.9	81	0.28 ± 0.27	114	6.0 ± 1.1	97	5.1 ± 1.4	67	5.0 ± 1.4
	Female	100	48.7 ± 2.7	84	0.29 ± 0.27	117	6.1 ± 1.0	99	5.1 ± 1.3	68	5.0 ± 1.3
2018	Male	70	49.3 ± 2.6	0		70	6.3 ± 0.8	70	5.6 ± 1.1	65	5.4 ± 1.0
	Female	67	49.4 ± 2.6	0		67	6.3 ± 0.7	67	5.5 ± 1.1	59	5.5 ± 1.0

*Note:* *N* is the number of individuals, mean and standard deviation (SD) is shown.

### 3.2 Relationship Between Current Reproduction and Current Physiology

**FIGURE 2:**
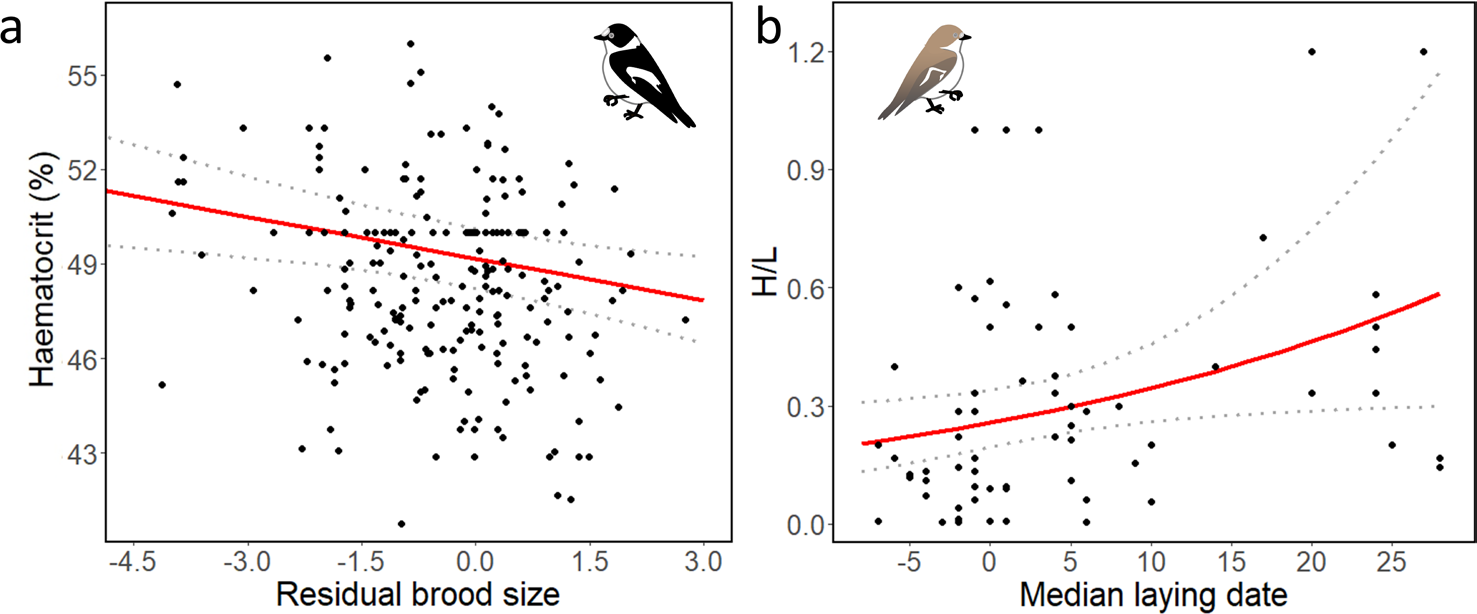
Relationships (with the 95% confidence intervals [dotted lines]) between current‐year reproduction and physiology during the nestling‐rearing period of collared flycatchers. (a) In males, the relationship between haematocrit during the nestling‐rearing period and regression residuals of brood size on median laying date; and (b) in females, the relationship between H/L ratio during the nestling‐rearing period and median laying date. The continuous red lines show the values predicted by the models.

In males, current brood size and haematocrit were negatively associated (*n* = 214, *χ* ^2^ = 7.9, *p* = 0.0049, Figure 2a), but there was no relationship between haematocrit and reproduction in females. The models for both sexes showed the same between‐year changes in haematocrit as the models investigating the between‐year and sex patterns of haematocrit (females: *n* = 223, *χ* ^2^ = 18.9, *p* = 0.0003; males: *n* = 214, *χ* ^2^ = 16.0, *p* = 0.00011). Current laying date and H/L ratio had a positive relationship in females (*n* = 66, *χ* ^2^ = 4.6, *p* = 0.033; Figure 2b) but there was no association between H/L ratio and reproduction in males; the model selection resulted in the null model.

### 3.3 Relationship Between Previous Reproduction and Current Physiological State

**FIGURE 3:**
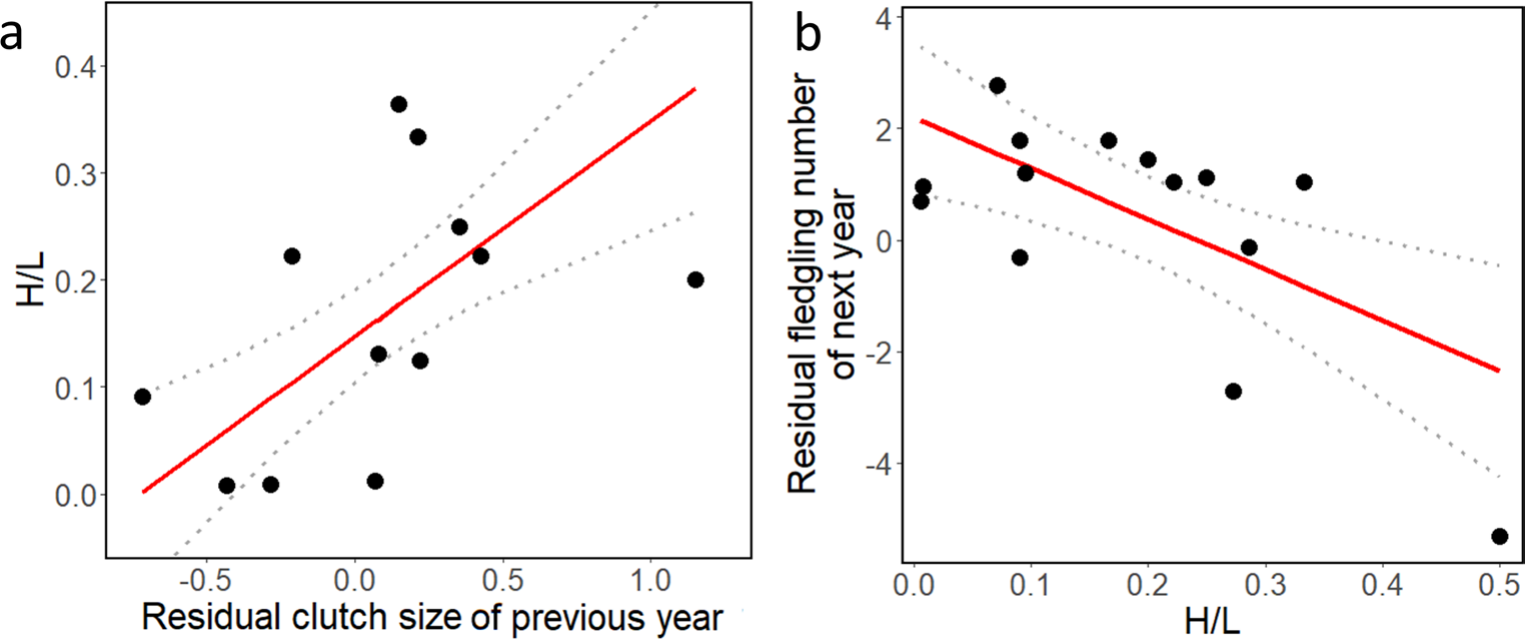
Carry‐over effects in relation to H/L ratio during the nestling‐rearing period in female collared flycatchers. Relationships (with the 95% confidence intervals [dotted lines]) between (a) regression residual of previous‐year clutch size on median laying date and current H/L ratio. (b) current H/L ratio and the regression residuals of the next‐year number of fledglings on median laying date. The continuous red lines show the predicted values predicted by the models.

There were no associations between past reproduction and current haematocrit in either sex, but in the case of males, current brood size showed the same pattern as the previous models, a negative association with haematocrit (*n* = 31, *χ* ^2^ = 4.2, *p* = 0.039). In females, previous year clutch size was positively associated with current H/L ratio (*n* = 12, *χ* ^2^ = 15.2, *p* < 0.0001; Figure 3a), suggesting that larger previous year clutch size was associated with poorer subsequent year physiological condition. In the final model, current laying date and H/L were positively associated in females (*n* = 12, *χ* ^2^ = 12.5, *p* < 0.001), the same pattern we showed above. In males, we found no significant patterns; the model selection resulted in the null model.

### 3.4 Relationship Between Current Physiology and Future Reproduction

**FIGURE 4:**
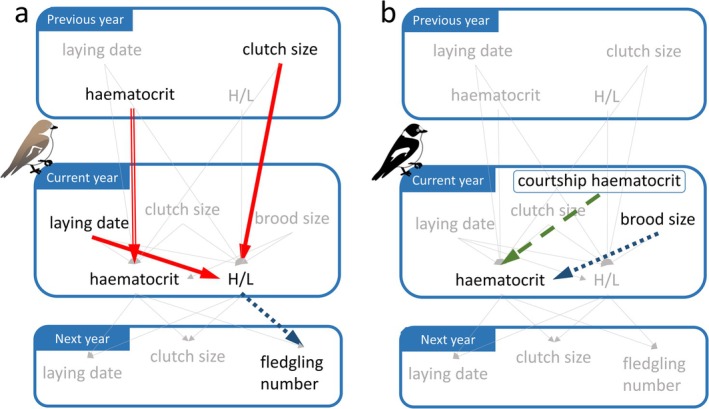
Summary of the associations between physiological states of different life phases, and relationships between reproduction and physiological state during nestling rearing in (a) female and (b) male collared flycatchers (Solid red arrow: positive relationship; dotted blue arrow: negative relationship; outlined red arrow: repeatability; dashed green arrow: decrease; thin solid grey arrow: no relationship).

For haematocrit, we detected no significant relationships with future reproductive variables in either sex, even in extended models, aside from expected (from the between‐year analysis of haematocrit) year effects (all *χ* ^2^ > 11.1, all *p* < 0.011, for further details about the models, see Appendix S3). In females, the current H/L ratio was negatively associated with the subsequent year's fledging success (*n* = 14, *χ* ^2^ = 9.7, *p* = 0.0018, Figure 3b). We calculated Cook's distance for a single datapoint that seemed to be an outlier. It was highly influential (Cook's *D* = 2), even though the H/L ratio of 0.5 was not an outlier by Grubbs test (*p* = 0.18). After removing this data point, the significant relationship disappeared; the model selection resulted in the null model (Table B8.5.x in Appendix S3). In males, we found no significant relationships between current physiology and subsequent year reproduction (model selection resulted in the null model). For an overview of the patterns we revealed, see Figure 4.

## 4 Discussion

### 4.1 Haematocrit

Haematocrit had low and not significant between‐year repeatability in both sexes. Year‐standardised haematocrit showed a moderate repeatability across consecutive years, with higher repeatability observed in females similarly to what Potti (2007) showed. This suggests that although haematocrit retains a degree of individual consistency across consecutive years (Boross et al. 2012), environmental factors have an increasing effect on haematocrit over a longer time span (Potti 2007). Given that collared flycatchers have a relatively short lifespan, this result implies that haematocrit may be a relatively stable physiological trait even over a significant portion of an individual's life. Additionally, females had higher haematocrit than males, which shows sex‐dependent patterns of haematocrit. From the view of sex‐dependent patterns, results from previous studies were mixed, as females had higher haematocrit than males in great tits (Hõrak, Jenni‐Eiermann, et al. 1998; Hõrak, Ots, and Murumägi 1998), but not in pied flycatchers (Potti 2007). In previous years in our study population, both patterns were present depending on the year (Boross et al. 2012; for review, see Fair, Whitaker, and Pearson 2007). Hormonal processes connected to egg‐laying, specifically receptor‐mediated effects of endogenous oestrogens, have a strong effect on haematocrit in females (Wagner et al. 2008), which may contribute to the differences between sexes that we found in our study. Females may need to have relatively high haematocrit levels during nestling rearing (Hõrak, Jenni‐Eiermann, et al. 1998, Hõrak, Ots, and Murumägi 1998) in multiple years, allocating less energy to other aspects of self‐maintenance.

We found that the mean haematocrit levels in our collared flycatcher population fluctuated between years. This aligns with previous studies that revealed the environmental dependency of haematocrit in the form of yearly fluctuations (Potti 2007; Boross et al. 2012). Food availability shows an interannual fluctuation at our study site (Török et al. 2004). Food availability influences haematocrit; it increases with additional food source (Hoi‐Leitner et al. 2001; Merino and Potti 1998; Santangeli et al. 2012), while it decreases during fasting (Boismenu et al. 1992; Merila and Svensson 1995). The fluctuations in food availability, therefore, likely contribute to the between‐year fluctuation of haematocrit.

The within‐individual (i.e., longitudinal) analyses of males confirmed that the haematocrit decreased from the courtship period to the nestling‐rearing period, which pattern is in concordance with the findings of a cross‐sectional analysis performed in the same population (Boross et al. 2012). We can see that the amount of decrease was highly variable across individuals (Figure 1). Elevated haematocrit levels during early breeding stages may reflect the high metabolic demands of migration, as suggested by Boross et al. (2012), which may help explain this pattern. Although haematocrit may rise due to erythropoiesis to react to heightened oxygen demand of physical exertion (Saino et al. 1997; Hõrak, Ots, and Murumägi 1998), it can also decline if there is not enough energy to upkeep erythropoiesis when energy is allocated to increased parental effort (Fowler and Williams 2017) or migration, resulting in the decrease of haematocrit (Owen and Moore 2006). We propose three alternative explanations: first, we suggest that elevated levels of haematocrit may be caused by dehydration (Vleck et al. 2000) in males during their courtship and nest box displaying behaviour, where they might not leave the area surrounding the nest box, therefore having limited access to water for a prolonged time. However, we did not investigate water availability. Second, we also suggest that avian malaria infection could be behind this pattern. *Haemoproteus* sp. infection decreases within male collared flycatchers in our study population between the courtship phase and the nestling‐rearing phase (Szöllősi et al. 2016). These parasites could destroy red blood cells, resulting in fewer red blood cells, thus lower haematocrit. In our blood samples, 6.5% of the plasma showed discoloration due to haemolysis, which is much lower than the 50% prevalence of *Haemoproteus* infection in the study population during the nestling phase (Szöllősi et al. 2016). Therefore, we cannot be sure that *Haemoproteus* infection causes this decrease of haematocrit. Finally, elevated haematocrit during courtship can be caused by increased testosterone levels associated with sperm production (Wingfield and Farner 1993). Experimentally increased testosterone causes elevated haematocrit levels in captive white‐plumed honeyeaters ( *Lichenostomus penicillatus* ) (Buttemer and Astheimer 2000), American kestrels ( *Falco sparverius* ) (Rehder et al. 1982), and Japanese quails ( *Coturnix japonica* ) (Kobayashi et al. 2022); however, haematocrit did not change after the experimental increase of testosterone in captive male yellow‐legged gulls ( *Larus cachinnans* ) (Alonso‐Alvarez et al. 2002).

With regard to the connections between reproduction and physiology, we found no evidence of COEs. When analysing current reproduction and physiology, we found that haematocrit levels were linked to the residual number of nestlings at the time of sampling in males, but we found no significant patterns in females. This supports that the decrease of haematocrit in males from courtship to nestling rearing may be caused by the high energetic demands of nestling rearing, as males had lower haematocrit levels than females during the nestling phase. Increased parental effort leads to lowered haematocrit levels in female common starlings ( *Sturnus vulgaris* ) (Fowler and Williams 2017), suggesting a trade‐off between haematocrit level and reproduction. Males may experience a trade‐off between self‐maintenance (maintaining haematocrit to have adequate oxygen‐carrying capacity) and parental care (provisioning, allocating resources to offsprings) each breeding season, as males raising larger broods have relatively lower haematocrit levels. In contrast, increased brood size resulted in higher haematocrit levels in great tits (Hõrak, Ots, and Murumägi 1998). The higher level of haematocrit in females suggests that irrespective of current reproductive effort, females may need to have relatively high haematocrit levels during nestling rearing in multiple years, allocating less energy to other aspects of self‐maintenance.

### 4.2 H/L Ratio

H/L ratio varies across avian families; in the *Muscicapoidea* family, where the collared flycatcher belongs, it is relatively low (0.37 on average) (Minias 2019). In a closely related species, the pied flycatcher, during nestling‐rearing, the average value of H/L in females is 0.42 (Moreno et al. 2002), while in males, it ranges from 0.34 to 0.62 (Ilmonen et al. 2003; Kerimov et al. 2017). In collared flycatchers, females' H/L ratio was 0.45 in the study population from 2001 to 2005 (Hargitai, Prechl, and Torok 2006; Hargitai, Mateo, and Torok 2010). In this study, the H/L ratio was 0.28 on average and did not change between years (2015–2016) (see Table 2 for values by year and sex), which is lower than the previously published values in our population. The H/L ratio is linked to parasitic infestation, longevity, sociality, inclement weather events, pollution and increased breeding effort in birds (Minias 2019); therefore, a combination of extrinsic and intrinsic factors may cause it to change in a 10‐year period, while it seems relatively stable in a 2–4 year period. As avian malaria infection is prevalent in our study population (Szöllősi et al. 2016), we cannot overlook the potential effects it may have on H/L ratio. Avian malaria (e.g., *Haemoproteus*, *Plasmodium*) infected passerines have higher H/L ratios (Wojczulanis‐Jakubas et al. 2012), but in some species, H/L ratio is not linked with avian malaria infection (Granthon and Williams 2017; Santiago‐Alarcon et al. 2020). In some cases, this relationship is mediated by body condition, as infected individuals in good condition may have a lower H/L ratio than uninfected birds in worse condition (Bustillo‐de La Rosa et al. 2022). Nevertheless, a stable H/L ratio may indicate that the prevalence of avian malaria stays the same in our population. Although we could not investigate the repeatability of H/L ratio due to limited sample size, the H/L ratio can be repeatable between life phases (Hõrak et al. 2002), which may help keep H/L ratio the same between years.

We found a significant positive relationship between H/L ratio and laying date in females, suggesting that individuals that started breeding later exhibited higher stress levels than those who started breeding earlier. The laying date conveys a lot of information about reproductive success in a breeding season, as it correlates with multiple reproductive variables. Individuals in poorer physiological conditions may delay breeding (Bêty et al. 2003; Marra and Holberton 1998), or late breeding may impose higher physiological stress due to limited food availability (Siikamäki 1998), leading to smaller reproductive success and increased H/L ratios. Age also determines the timing of breeding in female collared flycatchers in the Gotland population on a 32‐year long dataset (Martyka et al. 2023), as 3‐year‐old females start breeding the earliest.

We found that the H/L ratio is associated with future reproductive success in female collared flycatchers. In more detail, females with a lower H/L ratio during nestling‐rearing had more fledglings in the subsequent breeding season, suggesting a potential COE between these. We should be careful interpreting this result, since the pattern disappeared after removing an influential point. In the context of COEs of the H/L ratio on future breeding, studies that we know only showed relationships with return rate in the subsequent years. These results are mixed, as the H/L ratio during incubation did not affect return rate in eiders (Hanssen et al. 2003), but it did in fledgling pied flycatchers, as fledglings who returned to breed had lower H/L levels (Lobato et al. 2005). However, none of these studies addressed COEs of the H/L ratio during or after nestling rearing. There are few studies on CORT in this matter; effects of postbreeding CORT levels are also mixed, as there are no COEs on future reproductive output in eiders (Steenweg et al. 2022; Harms et al. 2015) and grey‐headed albatrosses (Crossin et al. 2017), but CORT did affect the timing of the arrival to the breeding site in eiders (Harms et al. 2015). Deferring black‐browed albatrosses and giant petrels had higher CORT levels after the previous breeding season (Crossin et al. 2013, 2017), but this pattern was not apparent in kittiwakes (Schultner et al. 2014). These differences in patterns might be due to species‐specific breeding ecology, wintering environmental conditions, such as food availability or the timing and duration of migration (Ramos et al. 2018; Schultner et al. 2014).

We also found a positive relationship between current clutch size and H/L ratios during the breeding season of the subsequent year in females. We showed that females with lower H/L ratios during nestling rearing‐initiated breeding earlier; additionally, the relative clutch size (as residual from a regression on laying date) was positively associated with future health state. These results suggest that the COEs between H/L ratio and reproduction may be bidirectional throughout multiple years, similar to the bidirectional relationship between reproduction and another stress index, the CORT: as experimentally increased CORT reduces fledging success in pied flycatchers (Silverin 1986), and increased parental effort results in higher CORT levels in starlings (Fowler and Williams 2017), but not in pied flycatchers (Ilmonen et al. 2003), although these studies were not assessing COEs. Males in our study exhibited no evidence of COEs with respect to the aspects of stress measured by H/L ratio, suggesting sex‐dependent COEs between physiology and reproduction. It is important to note that we analysed only the COEs of H/L ratio measured in 2016, which limits the generalisation of the revealed patterns. However, to our knowledge, our study is the first to show COEs of H/L ratio and reproduction in short‐lived birds. These findings illustrate how stress physiology and reproductive effort can be interconnected across life‐history stages, as both CORT and H/L can affect future reproduction. Although baseline CORT and H/L ratio are not correlated in previous studies (for review, see Davis and Maney 2018), similar patterns of COEs emerge between these two stress indices and reproduction.

## 5 Conclusions

Haematocrit levels fluctuate between years, most likely due to environmental causes, for example, food availability, while the H/L ratio remained at the same level in our population. Furthermore, females had higher haematocrit levels than males. Reproduction was linked to haematocrit in males and to H/L ratio in females, suggesting that different physiological traits may be important to maintain during reproduction in the two sexes. The observed sex differences may stem from the physiological changes and energetic demands associated with different functional roles during breeding (e.g., egg laying, sex‐specific hormonal responses). We found evidence that COEs between physiology and reproduction occur in a short‐lived, migratory species. Both previous and future reproductive effort were linked to the H/L ratio. However, these patterns were only apparent in females, suggesting that males have different physiological constraints on reproduction throughout their lifespan. Our results suggest that COEs between stress physiology and future reproduction may exhibit similar patterns across both short‐lived and long‐lived species. These patterns warrant further research of COEs of physiology and reproduction in passerine species.

## Author Contributions

**M. Laczi:** conceptualization (equal), data curation (equal), formal analysis (equal), investigation (equal), methodology (equal), supervision (equal), writing – original draft (equal), writing – review and editing (equal). **B. Rosivall:** investigation (equal), writing – review and editing (equal). **N. Boross:** investigation (equal), writing – review and editing (equal). **E. Szöllősi:** investigation (equal), writing – review and editing (equal). **D. Kötél:** investigation (equal), writing – review and editing (equal). **G. Hegyi:** investigation (equal), methodology (equal), writing – review and editing (equal). **G. Szabó:** conceptualization (equal), data curation (equal), formal analysis (equal), investigation (equal), methodology (equal), validation (equal), visualization (equal), writing – original draft (equal), writing – review and editing (equal). **E. Szász:** investigation (equal), writing – review and editing (equal). **M. Jablonszky:** investigation (equal), writing – review and editing (equal). **J. Török:** conceptualization (equal), funding acquisition (equal), investigation (equal), methodology (equal), project administration (equal), resources (equal), supervision (equal), writing – original draft (equal), writing – review and editing (equal). **K. Krenhardt:** investigation (equal), writing – review and editing (equal). **G. Markó:** investigation (equal), writing – review and editing (equal). **M. Herényi:** data curation (equal), investigation (equal), writing – review and editing (equal). **G. Nagy:** data curation (equal), investigation (equal), writing – review and editing (equal). **S. Zsebők:** investigation (equal), writing – review and editing (equal).

## Ethics Statement

We conducted this study with research permits from the regional nature conservation authority (permit numbers KTVF:10949‐8/2013, PE/KTF/11978‐5/2015, PE/KTF11978‐6/2015). We conform to relevant national guidelines and regulations.

## Conflicts of Interest

The authors declare no conflicts of interest.

## Supporting information


Appendix S1.

Appendix S2.

Appendix S3.

## Data Availability

Data and code for the analyses are provided as Appendices S1 and S2 files.
