# Supplementary material for: Sex‐Dependent Carry‐Over Effects Between Physiological State and Reproduction in a Passerine Species
Source: Ecol Evol. 2025 Jul 20;15(7):e71816. doi: 10.1002/ece3.71816 (PMC12277126; doi:10.1002/ece3.71816)
Supplement: Supplementary file 3 — Appendix S3. [file ECE3-15-e71816-s002.docx]

Appendix S3

# Appendix A – Parameters of correcting reproductive variables to median laying date

***Table A1*** *Parameters of the linear regressions fitted when calculating the relationship between laying date and clutch size, brood size and fledgling number. Number of eggs, number of nestlings at the time of taking blood sample from the parents and the number of fledged nestlings (reaching 13 days of age) were corrected for median laying date of the whole collared flycatcher population. We took the residuals of the fitted regressions as the corrected values.*

| year | response variable | R^2^ | p | n |
| --- | --- | --- | --- | --- |
| 2013 | number of eggs | 0.07 | 0.00067 | 173 |
|  | number of fledglings | 0.17 | 0.00083 | 64 |
| 2014 | number of eggs | 0.22 | p<0.0001 | 186 |
|  | number of nestlings | 0.05 | 0.24 | 29 |
|  | number of fledglings | 0.16 | 0.00047 | 72 |
| 2015 | number of eggs | 0.23 | p<0.0001 | 213 |
|  | number of nestlings | 0.50 | 0.00011 | 24 |
|  | number of fledglings | 0.30 | p<0.0001 | 113 |
| 2016 | number of eggs | 0.11 | p<0.0001 | 258 |
|  | number of nestlings | 0.13 | 0.00094 | 78 |
|  | number of fledglings | 0.13 | p<0.0001 | 134 |
| 2017 | number of eggs | 0.27 | p<0.0001 | 220 |
|  | number of fledglings | 0.16 | p<0.0001 | 178 |
| 2018 | number of eggs | 0.32 | p<0.0001 | 263 |
|  | number of nestlings | 0.06 | 0.15 | 38 |
|  | number of fledglings | 0.24 | p<0.0001 | 206 |
| 2019 | number of eggs | 0.14 | p<0.0001 | 333 |
|  | number of fledglings | 0.11 | p<0.0001 | 240 |

# Appendix B – GLM and GLMM information

The following tables provide information on the subset generalised linear models (GLMs) and generalised linear mixed models (GLMMs) created during the model selection process. The last models are the final versions used in the manuscript. For the description of our model selection approach, see the Materials and Methods section in the manuscript.

Description of the variables:

year: Sampling year of the physiological variable. Factor with the maximum of 4 levels (2014, 2015, 2016, 2018).

ID: Unique ID for each collared flycatcher.

sex: Sex of collared flycatchers

haem: Haematocrit value of the individuals. Numeric variable: the length of (blood-plasma)/blood in a capillary.

stdhaem: Haematocrit value of the individuals, standardised by years (mean=0, SD=1) separately for each sex

prev_haem: Haematocrit value of the individuals from the previous year. Numeric variable: the length of (blood-plasma)/blood in a capillary.

hperl: H/L ratio. Numeric variable: The ratio of heterophil granulocytes and leukocytes counted on blood smears.

prevhperl: H/L ratio from the previous year.

medLD: Median laying date. Numeric variable: The median laying date of the investigated collared flycatcher population in each year.

clutch_size: Number of eggs corrected for median laying date each year, as residuals from a linear regression. Numeric variable.

brood_size: Number of nestlings at the time of taking blood samples, corrected for median laying date each year, as residuals from a linear regression. Numeric variable.

fledglings: Number of nestlings reaching 13 days of age, corrected for median laying date each year, as residuals from a linear regression. Numeric variable.

prev_medLD: medLD of the bird from the previous year. Numeric variable.

prev_clutch_size: clutch_size from the previous year.

next_medLD: medLD of the next year

next_clutch_size: clutch size of the next year

next_fledglings: fledglings from the next year

The following operators can separate the fixed effects: “ + ”: no interaction between the variables, “:“: the interaction term of two variables.

Abbreviations:
AIC: Akaike Information Criterion
BIC: Bayesian Information Criterion

## B1 Between-year patterns of haematocrit models

**Table B1** Models for between year pattern of haematocrit. See Appendix B - GLMM information for the description of the table.

| Model number | Number of observation | Number of individuals (random factor) | AIC | BIC | Log-likelihood | Deviance | Residual degrees of freedom | Model formula |
| --- | --- | --- | --- | --- | --- | --- | --- | --- |
| 1 | 485 | 425 | -2120.929 | -2079.087 | 1070.464 | 0.1899343 | 477 | haem ~ year + sex + year:sex + (1\|ID) |
| 2 | 485 | 425 | -2124.89 | -2095.601 | 1069.445 | 0.1889294 | 480 | haem ~ year + sex + (1\|ID) |

## B2 Between-year patterns of H/L ratio models

**Table B2** Models for between year pattern of H/L ratio. See Appendix B - GLMM information for the description of the table.

| Model number | Number of observation | Number of individuals (random factor) | AIC | BIC | Log-likelihood | Deviance | Residual degrees of freedom | Model formula |
| --- | --- | --- | --- | --- | --- | --- | --- | --- |
| 1 | 202 | 194 | -102.1078 | -82.25822 | 57.05391 | 255.0405 | 198 | hperl ~ sex + year + sex:year + (1\|ID) |
| 2 | 202 | 194 | -103.8368 | -87.29549 | 56.91841 | 255.3342 | 199 | hperl ~ sex + year + (1\|ID) |
| 3 | 202 | 194 | -105.5283 | -92.29519 | 56.76413 | 255.669 | 200 | hperl ~ year + (1\|ID) |
| 4 | 202 | 194 | -106.7756 | -96.85083 | 56.38782 | 256.4872 | 201 | hperl ~ (1\|ID) |

## B3 Patterns of current reproduction and current haematocrit models

Three females with late median laying dates (series of Grubbs tests, all p<0.008) were removed from the analysis.

**Table B3.1** Models for patterns of current reproduction and current haematocrit of females. See Appendix B - GLMM information for the description of the table

| Model number | Number of observation | Number of individuals (random factor) | AIC | BIC | Log-likelihood | Deviance | Residual degrees of freedom | Model formula |
| --- | --- | --- | --- | --- | --- | --- | --- | --- |
| 1 | 223 | 201 | -965.6716 | -904.3425 | 500.8358 | 0.1222149 | 207 | haem ~ medLD +brood_size + clutch_size + year + medLD:year + brood_size:year + clutch_size:year + (1\|ID) |
| 2 | 223 | 201 | -968.7351 | -917.6275 | 499.3675 | 0.1309328 | 210 | haem ~ medLD + brood_size + clutch_size + year + brood_size:year + clutch_size:year + (1\|ID) |
| 3 | 223 | 201 | -970.6124 | -929.7263 | 497.3062 | 0.1216959 | 213 | haem ~ medLD + brood_size + year + clutch_size:year + (1\|ID) |
| 4 | 223 | 201 | -971.6099 | -940.9454 | 494.805 | 0.109707 | 216 | haem ~ medLD + brood_size + clutch_size + year + (1\|ID) |
| 5 | 223 | 201 | -973.6025 | -946.3451 | 494.8013 | 0.1109346 | 217 | haem ~ medLD + brood_size + year + (1\|ID) |
| 6 | 223 | 201 | -974.0058 | -950.1556 | 494.0029 | 0.09734364 | 218 | haem ~ medLD + year + (1\|ID) |
| 7 | 223 | 201 | -973.1091 | -952.6661 | 492.5546 | 0.09909755 | 219 | haem ~ year + (1\|ID) |

**Table B3.2** Models for patterns of current reproduction and current haematocrit of males. See Appendix B - GLMM information for the description of the table

| Model number | Number of observation | Number of individuals (random factor) | AIC | BIC | Log-likelihood | Deviance | Residual degrees of freedom | Model formula |
| --- | --- | --- | --- | --- | --- | --- | --- | --- |
| 1 | 214 | 191 | -917.2056 | -856.618 | 476.6028 | 0.02152172 | 198 | haem ~ medLD +brood_size + clutch_size + year + medLD:year + brood_size:year + clutch_size:year + (1\|ID) |
| 2 | 214 | 191 | -922.9897 | -872.5 | 476.4948 | 0.02128194 | 201 | haem ~ medLD + brood_size + clutch_size + year + medLD:year + brood_size:year + (1\|ID) |
| 3 | 214 | 191 | -928.2546 | -887.8629 | 476.1273 | 0.02383021 | 204 | haem ~ medLD + brood_size + clutch_size + medLD:year + (1\|ID) |
| 4 | 214 | 191 | -930.6844 | -900.3906 | 474.3422 | 0.0278554 | 207 | haem ~ medLD + brood_size + clutch_size + year + (1\|ID) |
| 5 | 214 | 191 | -926.8232 | -906.6273 | 469.4116 | 0.01785238 | 208 | haem ~ medLD + clutch_size + brood_size + (1\|ID) |
| 6 | 214 | 191 | -930.2835 | -906.7217 | 472.1418 | 0.02892146 | 209 | haem ~ brood_size + year + (1\|ID) |

## B4 Patterns of current reproduction and current H/L ratio models

**Table B4.1** Models for patterns of current reproduction and current H/L ratio of females. See Appendix B - GLMM information for the description of the table.

| Model number | Number of observation | AIC | BIC | Log-likelihood | Deviance | Residual degrees of freedom | Model formula |
| --- | --- | --- | --- | --- | --- | --- | --- |
| 1 | 66 | -19.20485 | -8.256578 | 14.60243 | 79.27831 | 62 | hperl ~ medLD + brood_size + clutch_size |
| 2 | 66 | -21.2039 | -12.44528 | 14.60195 | 79.2793 | 63 | hperl ~ medLD + brood_size |
| 3 | 66 | -22.92941 | -16.36045 | 14.46471 | 79.56416 | 64 | hperl ~ medLD |

**Table B4.2** Models for patterns of current reproduction and current H/L ratio of males. See Appendix B - GLMM information for the description of the table.

| Model number | Number of observation | AIC | BIC | Log-likelihood | Deviance | Residual degrees of freedom | Model formula |
| --- | --- | --- | --- | --- | --- | --- | --- |
| 1 | 62 | -24.34625 | -15.83771 | 16.17312 | 99.62908 | 58 | hperl ~ medLD+ brood_size + clutch_size |
| 2 | 62 | -24.40602 | -15.89749 | 16.20301 | 99.54895 | 59 | hperl ~ medLD+ clutch_size |
| 3 | 62 | -26.19816 | -19.81676 | 16.09908 | 99.82786 | 60 | hperl ~ medLD |
| 4 | 62 | -27.98782 | -23.73356 | 15.99391 | 100.1108 | 61 | hperl ~ 1 |

## B5 Patterns of previous reproduction and current H/L ratio models

Two females and one male had late median laying dates, three females had outlier H/L ratio, , two males had late median laying dates in the previous year (confirmed by Grubbs-tests, all p<0.070).

**Table B5.1** Models for patterns of previous reproduction and current H/L ratio of females. See Appendix B - GLMM information for the description of the table

| Model number | Number of observation | AIC | BIC | Log-likelihood | Deviance | Residual degrees of freedom | Model formula |
| --- | --- | --- | --- | --- | --- | --- | --- |
| 1 | 12 | -19.59676 | -16.68732 | 15.79838 | 0.05048729 | 7 | hperl ~ medLD + brood_size + prev_medLD + prev_clutch_size |
| 2 | 12 | -20.83244 | -18.40791 | 15.41622 | 0.05380761 | 8 | hperl ~ medLD + prev_medLD + prev_clutch_size |
| 3 | 12 | -21.66386 | -19.72423 | 14.83193 | 0.0593111 | 9 | hperl ~ medLD + prev_clutch_size |

**Table B5.2** Models for patterns of previous reproduction and current H/L ratio of males. See Appendix B - GLMM information for the description of the table

| Model number | Number of observation | AIC | BIC | Log-likelihood | Deviance | Residual degrees of freedom | Model formula |
| --- | --- | --- | --- | --- | --- | --- | --- |
| 1 | 14 | -0.6648289 | 3.169515 | 6.332414 | 0.3317245 | 9 | hperl ~ medLD + brood_size + prev_medLD + prev_clutch_size |
| 2 | 14 | -2.3509 | 0.8443862 | 6.17545 | 0.339247 | 10 | hperl ~ medLD + brood_size + prev_clutch_size |
| 3 | 14 | -4.149984 | -1.593755 | 6.074992 | 0.3441507 | 11 | hperl ~ medLD + prev_clutch_size |
| 4 | 14 | -5.686531 | -3.769359 | 5.843265 | 0.355734 | 12 | hperl ~ prev_clutch_size |
| 5 | 14 | -4.708891 | -3.430776 | 4.354446 | 4.354446 | 13 | hperl ~ 1 |

## B6 Patterns of previous reproduction and current haematocrit models

Two females had late median laying dates in the previous year, and two males had late median laying dates (both Grubbs-tests p<0.001), and were excluded from the analysis.

**Table B6.1** Models for patterns of previous reproduction and current haematocrit of females. See Appendix B - GLMM information for the description of the table

| Model number | Number of observation | AIC | BIC | Log-likelihood | Deviance | Residual degrees of freedom | Model formula |
| --- | --- | --- | --- | --- | --- | --- | --- |
| 1 | 38 | -161.4417 | -146.7035 | 89.72086 | 0.01979271 | 30 | haem ~ medLD + brood_size + prev_medLD + prev_clutch_size + year + prev_medLD:year + prev_clutch_size:year |
| 2 | 38 | -163.1882 | -150.0875 | 89.5941 | 0.01992521 | 31 | haem ~ medLD + brood_size + prev_medLD + prev_clutch_size + year + prev_medLD:year |
| 3 | 38 | -163.6821 | -152.219 | 88.84107 | 0.02073077 | 32 | haem ~ medLD + brood_size + prev_medLD + year + prev_clutch_size |
| 4 | 38 | -165.6809 | -155.8557 | 88.84044 | 0.020733145 | 33 | haem ~ medLD + brood_size + prev_medLD + year |
| 5 | 38 | -167.6766 | -159.4887 | 88.8383 | 0.02073379 | 34 | haem ~ medLD + brood_size + prev_medLD |
| 6 | 38 | -167.9837 | -161.4333 | 87.99184 | 0.02167837 | 35 | haem ~ medLD + prev_medLD |
| 7 | 38 | -168.8504 | -163.9376 | 87.4252 | 0.02233462 | 36 | haem ~ medLD |
| 8 | 38 | -168.83 | -165.5548 | 86.41501 | 0.02355425 | 37 | haem ~ 1 |

**Table B6.2** Models for patterns of previous reproduction and current haematocrit of males. See Appendix B - GLMM information for the description of the table. Current haematocrit and brood size are negatively correlated (n=31, χ2=4.2, p=0.039).

| Model number | Number of observation | AIC | BIC | Log-likelihood | Deviance | Residual degrees of freedom | Model formula |
| --- | --- | --- | --- | --- | --- | --- | --- |
| 1 | 31 | -133.158 | -120.2521 | 75.57898 | 0.01384331 | 23 | haem ~ medLD + brood_size + prev_medLD + prev_clutch_size + year + prev_medLD:year + prev_clutch_size:year |
| 2 | 31 | -135.1577 | -123.6858 | 75.57884 | 0.01384344 | 24 | haem ~ medLD + brood_size + prev_medLD + prev_clutch_size + year + prev_medLD:year |
| 3 | 31 | -136.032 | -125.9941 | 75.01598 | 0.01435538 | 25 | haem ~ medLD + brood_size + prev_medLD + year + prev_clutch_size |
| 4 | 31 | -138.0191 | -129.4152 | 75.00955 | 0.01436133 | 26 | haem ~ medLD + brood_size + year + prev_clutch_size |
| 5 | 31 | -139.9418 | -132.7719 | 74.97092 | 0.01439718 | 27 | haem ~ medLD + brood_size + prev_clutch_size |
| 6 | 31 | -141.7022 | -135.9663 | 74.85111 | 0.01450889 | 28 | haem ~ prev_clutch_size + brood_size |
| 7 | 31 | -140.0114 | -135.7094 | 73.00569 | 0.01634334 | 29 | haem ~ brood_size |

## B7 Patterns of previous reproduction, haematocrit and current haematocrit models

**Table B7.1** Models for patterns of previous reproduction, haematocrit and current haematocrit of females. See Appendix B - GLMM information for the description of the table. In females, current haematocrit was positively associated with previous haematocrit (n=8, χ2=8.1, p=0.0045).

| Model number | Number of observation | AIC | BIC | Log-likelihood | Deviance | Residual degrees of freedom | Model formula |
| --- | --- | --- | --- | --- | --- | --- | --- |
| 1 | 8 | -31.975 | -31.41891 | 22.9875 | 0.001495477 | 2 | haem ~ prev_haem + medLD + brood_size + prev_medLD + prev_clutch_size |
| 2 | 8 | -33.58356 | -33.10691 | 22.79178 | 0.00157047 | 3 | haem ~ prev_haem + medLD + brood_size + prev_medLD |
| 3 | 8 | -35.1274 | -34.73019 | 22.5637 | 0.00166262 | 4 | haem ~ prev_haem + brood_size + prev_medLD |
| 4 | 8 | -35.1274 | -34.73019 | 22.5637 | 0.00166262 | 4 | haem ~ prev_haem + prev_medLD |
| 5 | 8 | -35.17657 | -34.93824 | 20.58828 | 0.002724401 | 6 | haem ~ prev_haem |

**Table B7.2** Models for patterns of previous reproduction, haematocrit and current haematocrit of males. See Appendix B - GLMM information for the description of the table. In males, current brood size and haematocrit were negatively associated (n=10, χ2= 8.7, p=0.0031)

| Model number | | Number of observation | AIC | BIC | Log-likelihood | Deviance | Residual degrees of freedom | Model formula |
| --- | --- | --- | --- | --- | --- | --- | --- | --- |
| 1 | 10 | | -51.45647 | -49.33838 | 32.72824 | 0.0008409829 | 4 | haem ~ prev_haem + medLD + brood_size + prev_medLD + prev_clutch_size |
| 2 | 10 | | -53.33776 | -51.52224 | 32.66888 | 0.0008510263 | 5 | haem ~ prev_haem + brood_size + prev_medLD + prev_clutch_size |
| 3 | 10 | | -54.35006 | -52.83714 | 32.17503 | 0.0009393726 | 6 | haem ~ prev_haem + brood_size + prev_clutch_size |
| 4 | 10 | | -54.81147 | -53.60113 | 31.40574 | 0.001095615 | 7 | haem ~ prev_haem + brood_size |
| 5 | 10 | | -53.73096 | -52.82321 | 29.86548 | 0.00149088 | 8 | haem ~ brood_size |

## B8 Patterns of current physiology and future reproduction models

One male and female had outlier H/L ratio (Grubbs-tests, both p<0.013), and 1 male and 2 female had late median laying dates in the next year (Grubbs-tests, all p>0.006), they were removed from the analyses

**Table B8.1** Models for patterns of current physiology and future median laying date of females. See Appendix B - GLMM information for the description of the table

| Model number | Number of observation | AIC | BIC | Log-likelihood | Deviance | Residual degrees of freedom | Model formula |
| --- | --- | --- | --- | --- | --- | --- | --- |
| 1 | 18 | 90.489 | 94.05049 | -41.2445 | 103.0503 | 15 | next_medLD ~ haem + hperl |
| 2 | 18 | 88.49252 | 91.16364 | -41.24626 | 103.0704 | 16 | next_medLD ~ haem |
| 3 | 18 | 86.65413 | 88.43488 | -41.32707 | 104 | 17 | next_medLD ~ 1 |

**Table B.8.2** Models for patterns of current physiology and future median laying date of males. See Appendix B - GLMM information for the description of the table

| Model number | Number of observation | AIC | BIC | Log-likelihood | Deviance | Residual degrees of freedom | Model formula |
| --- | --- | --- | --- | --- | --- | --- | --- |
| 1 | 18 | 77.79507 | 81.35656 | -34.89754 | 50.90692 | 15 | next_medLD ~ haem + hperl |
| 2 | 18 | 75.80199 | 78.47311 | -34.901 | 50.92648 | 16 | next_medLD ~ hperl |
| 3 | 18 | 73.80834 | 75.58908 | -34.90417 | 50.94444 | 17 | next_medLD ~ 1 |

**Table B8.3** Models for patterns of current physiology and future clutch size of females. See Appendix B - GLMM information for the description of the table

| Model number | Number of observation | AIC | BIC | Log-likelihood | Deviance | Residual degrees of freedom | Model formula |
| --- | --- | --- | --- | --- | --- | --- | --- |
| 1 | 17 | 31.6 | 34.93285 | -11.8 | 3.989123 | 14 | next_clutch_size ~ haem + hperl |
| 2 | 17 | 29.9359 | 32.43554 | -11.96795 | 4.068728 | 15 | next_clutch_size ~ hperl |
| 3 | 17 | 29.12442 | 30.79085 | -12.56221 | 4.363365 | 16 | next_clutch_size ~ 1 |

**Table B8.4** Models for patterns of current physiology and future clutch size of males. See Appendix B - GLMM information for the description of the table

| Model number | Number of observation | AIC | BIC | Log-likelihood | Deviance | Residual degrees of freedom | Model formula |
| --- | --- | --- | --- | --- | --- | --- | --- |
| 1 | 18 | 44.25344 | 47.81493 | -18.12672 | 7.897726 | 15 | next_clutch_size ~ haem + hperl |
| 2 | 18 | 42.2566 | 44.92771 | -18.1283 | 7.899111 | 16 | next_clutch_size ~ haem |
| 3 | 18 | 41.07127 | 42.85202 | -18.53564 | 8.264836 | 17 | next_clutch_size ~ 1 |

**Table B8.5** Models for patterns of current physiology and fledgling number of females. See Appendix B - GLMM information for the description of the table

| Model number | Number of observation | AIC | BIC | Log-likelihood | Deviance | Residual degrees of freedom | Model formula |
| --- | --- | --- | --- | --- | --- | --- | --- |
| 1 | 15 | 62.16151 | 64.99371 | -27.08076 | 32.49027 | 12 | next_fledglings ~ haem + hperl |
| 2 | 15 | 60.91036 | 63.03451 | -27.45518 | 34.15346 | 13 | next_fledglings ~ hperl |

**Table B8.5.x** Models for patterns of current physiology and fledgling number of females, after removing the influential point. See Appendix B - GLMM information for the description of the table

| Model number | Number of observation | AIC | BIC | Log-likelihood | Deviance | Residual degrees of freedom | Model formula |
| --- | --- | --- | --- | --- | --- | --- | --- |
| 1 | 14 | 50.74216 | 53.29839 | -21.37108 | 17.3604 | 11 | next_fledglings ~ haem + hperl |
| 2 | 14 | 49.44289 | 51.36006 | -21.72144 | 18.25144 | 12 | next_fledglings ~ hperl |
| 3 | 14 | 49.4975 | 50.7756 | -22.74875 | 21.1365 | 13 | next_fledglings ~ 1 |

**Table B8.6** Models for patterns of current physiology and fledgling number of males. See Appendix B - GLMM information for the description of the table

| Model number | Number of observation | AIC | BIC | Log-likelihood | Deviance | Residual degrees of freedom | Model formula |
| --- | --- | --- | --- | --- | --- | --- | --- |
| 1 | 16 | 38.60052 | 41.69087 | -15.30026 | 6.342317 | 13 | next_fledglings ~ haem + hperl |
| 2 | 16 | 38.99162 | 41.30939 | -16.49581 | 7.364623 | 14 | next_fledglings ~ hperl |
| 3 | 16 | 37.49438 | 39.03956 | -16.74719 | 7.599712 | 15 | next_fledglings ~ 1 |

## B9 Patterns of current haematocrit and future reproduction (extended) models

The predictors are haematocrit, year and their interaction. In this case, haematocrit and year were not independent predictors, so we centered haematocrit (haem_cent) by years and sexes.

**Table B9.1** Models for patterns of current haematocrit and future median laying date of females. See Appendix B - GLMM information for the description of the table

| Model number | Number of observation | AIC | BIC | Log-likelihood | Deviance | Residual degrees of freedom | Model formula |
| --- | --- | --- | --- | --- | --- | --- | --- |
| 1 | 101 | 649.453 | 672.9891 | -315.7265 | 3069.448 | 93 | next_medLD ~ year + haem_cent + year:haem_cent |
| 2 | 101 | 644.4825 | 660.1732 | -316.2412 | 3100.896 | 96 | next_medLD ~ year + haem_cent |
| 3 | 101 | 642.7302 | 650.5755 | -318.3651 | 3234.089 | 99 | next_medLD ~ haem |
| 4 | 101 | 643.342 | 648.5722 | -319.671 | 3318.812 | 100 | next_medLD ~ 1 |

**Table B9.2** Models for patterns of current haematocrit and future clutch size of females. See Appendix B - GLMM information for the description of the table

| Model number | Number of observation | AIC | BIC | Log-likelihood | Deviance | Residual degrees of freedom | Model formula |
| --- | --- | --- | --- | --- | --- | --- | --- |
| 1 | 100 | 203.0857 | 226.5322 | -92.54284 | 37.26855 | 92 | next_clutch_size ~ year + haem_cent + year:haem_cent |
| 2 | 100 | 197.2578 | 212.8888 | -92.62889 | 37.33275 | 95 | next_clutch_size ~ year + haem_cent |
| 3 | 100 | 195.4959 | 208.5218 | -92.74797 | 37.42177 | 96 | next_clutch_size ~ year |

**Table B9.3** Models for patterns of current haematocrit and fledgling number of females. See Appendix B - GLMM information for the description of the table

| Model number | Number of observation | AIC | BIC | Log-likelihood | Deviance | Residual degrees of freedom | Model formula |
| --- | --- | --- | --- | --- | --- | --- | --- |
| 1 | 79 | 271.5308 | 292.8558 | -126.7654 | 114.5282 | 71 | next_fledglings ~ year + haem_cent + year:haem_cent |
| 2 | 79 | 266.4144 | 280.6311 | -127.2072 | 115.8164 | 74 | next_fledglings ~ year + haem_cent |
| 3 | 79 | 267.1669 | 279.0141 | -128.5834 | 119.9227 | 75 | next_fledglings ~ year |

**Table B9.4** Models for patterns of current haematocrit and future median laying date of males. See Appendix B - GLMM information for the description of the table

| Model number | Number of observation | AIC | BIC | Log-likelihood | Deviance | Residual degrees of freedom | Model formula |
| --- | --- | --- | --- | --- | --- | --- | --- |
| 1 | 90 | 532.2106 | 554.7088 | -257.1053 | 1596.203 | 82 | next_medLD ~ year + haem_cent + year:haem_cent |
| 2 | 90 | 527.7805 | 542.7793 | -257.8902 | 1624.291 | 85 | next_medLD ~ year + haem_cent |
| 3 | 90 | 526.6273 | 539.1264 | -258.3137 | 1639.647 | 86 | next_medLD ~ year |

**Table B9.5** Models for patterns of current haematocrit and future clutch size of males. See Appendix B - GLMM information for the description of the table

| Model number | Number of observation | AIC | BIC | Log-likelihood | Deviance | Residual degrees of freedom | Model formula |
| --- | --- | --- | --- | --- | --- | --- | --- |
| 1 | 90 | 201.2199 | 223.7182 | -91.60996 | 40.35476 | 82 | next_clutch_size ~ year + haem_cent + year:haem_cent |
| 2 | 90 | 195.6526 | 210.6515 | -91.8263 | 40.54924 | 85 | next_clutch_size ~ year + haem_cent |
| 3 | 90 | 194.2826 | 206.7817 | -92.14131 | 40.83409 | 86 | next_clutch_size ~ year |

**Table B9.6** Models for patterns of current haematocrit and fledgling number of males. See Appendix B - GLMM information for the description of the table

| Model number | Number of observation | AIC | BIC | Log-likelihood | Deviance | Residual degrees of freedom | Model formula |
| --- | --- | --- | --- | --- | --- | --- | --- |
| 1 | 74 | 229.7422 | 250.4788 | -105.8711 | 75.75995 | 66 | next_fledglings ~ year + haem_cent + year:haem_cent |
| 2 | 74 | 223.9446 | 237.7689 | -105.9723 | 75.96735 | 69 | next_fledglings ~ year + haem_cent |
| 3 | 74 | 222.2511 | 233.7714 | -106.1256 | 76.28271 | 70 | next_fledglings ~ year |

# Appendix C - correlation matrices of breeding variables

**Table C1** Pearson-correlation of parameters that are predictors in the models, on the full dataset of female collared flycatchers. For names of variables, see Appendix B. R values are in the upper-triangle, p-values in the lower triangle.

|  | medLD | clutch_size | brood_size | prev_medLD | prev_clutch_size |
| --- | --- | --- | --- | --- | --- |
| medLD | - | -0.03 | -0.004 | 0.16 | -0.10 |
| clutch_size | 0.58 | - | 0.44 | 0.036 | 0.23 |
| brood_size | 0.95 | <0.0001 | - | 0.25 | -0.01 |
| prev_medLD | 0.13 | 0.74 | 0.031 | - | -0.22 |
| prev_clutch_size | 0.37 | 0.032 | 0.94 | 0.045 | - |

**Table C2** Pearson-correlation of parameters that are predictors in the models, on the full dataset of male collared flycatchers. For names of variables, see Appendix B. R values are in the upper-triangle, p-values in the lower triangle.

|  | medLD | clutch_size | brood_size | prev_medLD | prev_clutch_size |
| --- | --- | --- | --- | --- | --- |
| medLD | - | -0.15 | -0.04 | 0.023 | 0.12 |
| clutch_size | 0.016 | - | 0.52 | 0.061 | -0.02 |
| brood_size | 0.52 | <0.0001 | - | 0.13 | -0.14 |
| prev_medLD | 0.85 | 0.60 | 0.27 | - | -0.05 |
| prev_clutch_size | 0.31 | 0.88 | 0.24 | 0.70 | - |
